# Antigenic mapping of an H9N2 avian influenza virus reveals two discrete antigenic sites and a novel mechanism of immune escape

**DOI:** 10.1038/srep18745

**Published:** 2016-01-07

**Authors:** Thomas Peacock, Kolli Reddy, Joe James, Beata Adamiak, Wendy Barclay, Holly Shelton, Munir Iqbal

**Affiliations:** 1 The Pirbright Institute, Pirbright, Woking, UK; 2 Imperial College London, UK

## Abstract

H9N2 avian influenza virus is a major cause of poultry production loss across Asia leading to the wide use of vaccines. Efficacy of vaccines is often compromised due to the rapid emergence of antigenic variants. To improve the effectiveness of vaccines in the field, a better understanding of the antigenic epitopes of the major antigen, hemagglutinin, is required. To address this, a panel of nine monoclonal antibodies were generated against a contemporary Pakistani H9N2 isolate, which represents a major Asian H9N2 viral lineage. Antibodies were characterized in detail and used to select a total of 26 unique ‘escape’ mutants with substitutions across nine different amino acid residues in hemagglutinin including seven that have not been described as antigenic determinants for H9N2 viruses before. Competition assays and structural mapping revealed two novel, discrete antigenic sites “H9-A” and “H9-B”. Additionally, a second subset of escape mutants contained amino acid deletions within the hemagglutinin receptor binding site. This constitutes a novel method of escape for group 1 hemagglutinins and could represent an alternative means for H9N2 viruses to overcome vaccine induced immunity. These results will guide surveillance efforts for arising antigenic variants as well as evidence based vaccine seed selection and vaccine design.

Avian influenza virus, subtype H9N2 is enzootic in poultry across large areas of Asia, the Middle East, and North Africa where it imposes a large economic burden due to reduced growth rates in broilers and reduced fertility and egg production in breeders and layers^1^,^2^. Although H9 viruses are classified as low pathogenicity avian influenza viruses (LPAI), mortality in the field has been recorded of more than 50% and there have been instances of field strains displaying a highly pathogenic phenotype both in the field and in the laboratory^3^,^4^. H9N2 viruses also pose a threat to global human health both as a zoonotic agent in their own right, human infections have been reported in Hong Kong, across China, Bangladesh and Egypt^5^,^6^,^7^,^8^, but also as a donor of genes to other zoonotic avian influenza viruses such as the 1997 Hong Kong H5N1 outbreak, and the recent Chinese H7N9 and H10N8 outbreaks^9^,^10^,^11^. Recently it has been suggested the most effective method of preventing new zoonotic avian influenza subtypes from entering the human population would be better control of H9N2 viruses in poultry^12^.

Vaccination, as a means to reduce the impact of H9N2 viruses in poultry, has been adopted by many countries when the disease has become endemic^1^,^13^,^14^. However, in recent years vaccine failure in many areas has become commonplace due to the emergence of antigenic variants which harbour mutations in the major influenza antigen, hemagglutinin (HA)^15^,^16^,^17^,^18^. Consequently, efforts have been made to identify molecular markers in the HA gene that influence the antigenic diversity of H9N2 viruses leading to vaccine failure. All previous studies to this point have examined the antigenic architecture of the HA of H9N2 strains of the Bei/Y280-like lineage, the predominant lineage circulating in mainland China^19^,^20^,^21^,^22^,^23^. Here, however, we set out to investigate the antigenically distinct and more globally widespread group of H9N2 viruses found in poultry, the G1-like lineage, found across much of South East Asia, the Middle East and North Africa^3^,^6^,^24^,^25^,^26^,^27^,^28^,^29^,^30^,^31^,^32^.

In this study we describe a novel and efficient method of producing HA specific monoclonal antibodies in mice (mAbs). Analysis of a panel of mAbs directed against H9 HA protein revealed two discrete antigenic sites in the HA. We also confirm the antigenic importance of previously established H9 antigenic residues, positions 183 and 212, in the context of a contemporary G1-like virus^20^,^21^,^22^. In addition we also present a group of escape mutants with unique deletions within the 220 loop of the receptor binding site which have not been reported before in group 1 hemagglutinins of influenza A^33^,^34^.

## Results

### Neutralizing mAbs recognise one of two discrete antigenic sites

Hybridomas were screened by ELISA against purified UDL1/08 virus for secretion of H9HA specific binding mAbs. Nine positive clones were taken on for further characterization (Table 1).

The nine mAbs (CG12, EC12, HA9, JF7, JF8, IB3, ID2, HD8 and IG10) were purified and first tested to determine if they recognised a linear or conformational site. This was achieved by assessing whether the mAbs detected SDS-denatured, β-mercaptoethanol reduced recombinant UDL1/08 HA on Western blot (see supplementary Fig. S1 online). CG12, EC12, HA9 and JF8 did not give a positive band on Western blot whereas JF7, IB3, ID2, IG10, and HD8 did indicating recognition by the latter group of a linear epitope. Isotyping showed all mAbs were IgG2a with kappa light chain with the exception of ID2 which was IgG2b.

To assess the ability of the mAbs to neutralize virus, hemagglutinin inhibition (HI) and microneutralization (MNT) assays were performed with 0.1 mg/ml of mAb against the homologous virus UDL1/08 (Table 1). Two of the mAbs (IG10 and HD8) had no HI or MNT activity suggesting they were not targeting a neutralizing antigenic site and were subsequently not carried forward for further analysis. JF8 and ID2 displayed robust HI ability but gave non-concentration dependant, inconsistent MNT titres. Finally those remaining seven mAbs found to have neutralizing activity were analyzed biophysically through epitope binning and determination of the dissociation constant (K_D_) to purified recombinant HA. Epitope binning analysis indicated that the mAbs bound one of two non-overlapping antigenic sites that we designated H9-A (to which CG12, EC12, HA9, and JF7 bound) and H9-B (where JF8, ID2, and IB3 binding was observed). K_D_ values ranged between 5–96 nM, consistent with previous characterization of mouse mAbs to HA^35^.

### Antigenic sites H9-A and H9-B accurately reflect regions recognised by the chicken post-infection polyclonal response post-infection

**Figure 1:**
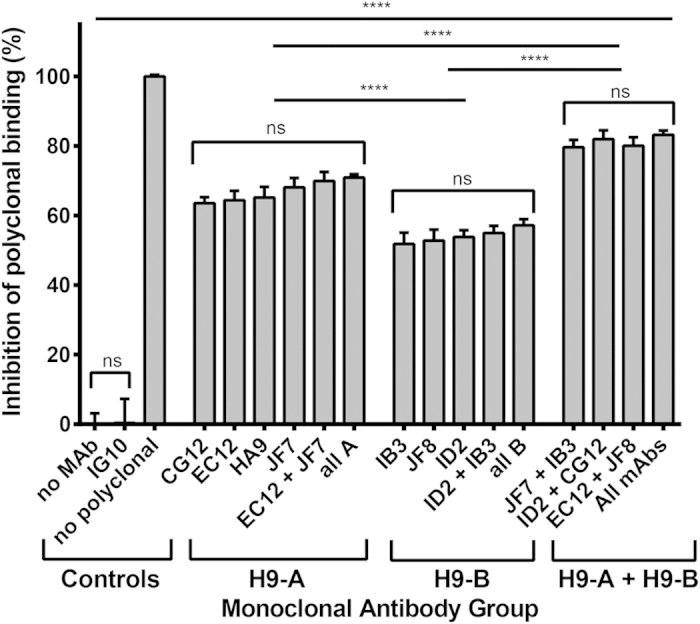
A competitive ELISA assay was performed to determine the percentage inhibition of chicken polyclonal antisera binding by mouse monoclonal antibodies. Different combinations of mouse mAbs were used to saturate all binding sites of purified UDL1/08 virus which was then probed with chicken polyclonal antisera. mAbs are grouped by proposed antigenic site bound, either H9-A or H9-B. 0% and 100% are set by using no mAbs and no polyclonal antisera controls respectively. IG10, a non-neutralizing mAb is used as an additional negative control. Statistical analysis was performed using a One-way Anova, **** signifies P ≤ 0.0001, ns shows p ≥ 0.05.

Although the use of mouse monoclonal antibodies has become a common way to antigenically map both human and avian strains of influenza, little has been done to investigate the relevance of mouse monoclonal antibodies to chicken polyclonal antisera. To address this we performed a competition ELISA. First the antigenic epitopes recognised by the mAbs were saturated on purified virus by adding an excess of the mouse mAbs before assessing the percentage of binding lost by chicken post-infection polyclonal antisera. The competition assay revealed that all the mouse mAbs, with the exception of the non-neutralising control, IG10, efficiently inhibited polyclonal chicken antisera binding and were therefore binding biologically relevant epitopes (Fig. 1). MAbs binding to site H9-A (CG12, EC12, HA9, and JF7) inhibited polyclonal antisera binding by an average of 64.3% whilst binders to the H9-B site (IB3, JF8, and ID2) inhibited binding by an average of 52.8% (Fig. 1). In contrast, IG10, one of the non-neutralizing mAbs, inhibited binding by an average of 0.3% which was not significantly higher than the no-monoclonal antibody control.

The results also support the evidence that proposed antigenic sites H9-A and H9-B constitute a pair of discrete non-overlapping epitopes. Addition of multiple antibodies from the same antigenic grouping did not increase inhibition (‘EC12 + JF7′, ‘all A’, ‘ID2 + IB3′, and ‘all B’), however adding mAbs from group A or B together resulted in significantly higher blocking of chicken polyclonal antisera (‘JF7 + IB3′, ‘ID2 + CG12′, ‘EC12 + JF8′, and ‘all mAbs’) (Fig. 1).

### Selection of monoclonal antibody escape mutants with amino acid substitutions

After several independent rescues from the same reverse genetics plasmids, UDL1/08 virus stocks were grown in eggs. Egg stocks were incubated with an excess (>1 mg/ml) of each mAb and then inoculated onto MDCK cells or eggs. The resulting infectious virus was then clonally selected in cells or eggs by limiting dilution. The virus HA gene was sequenced and compared to the sequenced starting material. Several different UDL1/08 virus stocks, independently rescued and grown, were tested in this way with each mAb to maximize discovery of different escape mutants. Sequence confirmed mutations were then engineered, using site directed mutagenesis, back into the UDL1/08 H9HA reverse genetics plasmids and rescued in conjunction with paired UDL1/08 N2NA plasmids onto a PR8 backbone. Reverse engineered mutant viruses were then tested by HI against the selecting mAb to confirm escape (Table 2).

**Figure 2:**
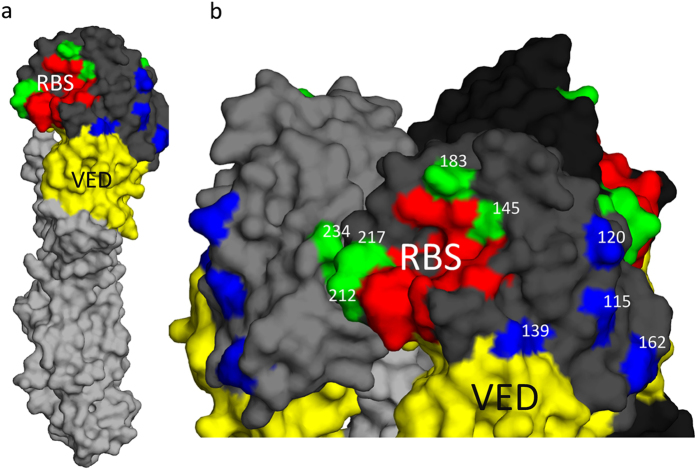
Structural locations of antigenic sites H9-A and H9-B. HA head domain in dark grey, HA stalk in Light grey, H9-A labelled in green, H9-B labelled in blue, receptor binding site (RBS) labelled in red and vestigial esterase domain (VED) labelled in yellow. Shown are HA monomer (**A**) and trimer of HA1 head domain (**B**) (PDB ID 1JSD). Numbers in panel B refer to H9 mature protein numbering. Residues highlighted in the receptor binding site are P92, G128, T129, S130, S131, A132, W142, N173, V180, L184, Y185, N214, G215, L216, G218, and R219.

Following sequencing of the escape mutant viruses it was clear they fell into two groups, those which escaped by single amino acid substitutions and those with deletions of 4–7 amino acids. The single amino acid escape mutants fell into two further groups, five mutated residues (145, 183, 212, 217, and 234, H9 mature protein numbering used throughout) were found from the H9-A binding mAbs (EC12, CG12, HA9, and JF7) whilst four different residues (115, 120, 139, and 162) were found mutated in viruses that escaped H9-B binding mAbs (IB3, ID2, and JF8) (Table 2). Following mapping of these residues onto the structure of H9HA it was clear that the residues allowing escape from the two distinct groups of mAbs cluster into two discrete regions with the H9-A residues surrounding the receptor binding site and the H9-B residues clustering just above the vestigial esterase domain (Fig. 2). None of the escape mutants found in this study disrupted or introduced predicated N-linked glycosylation sites as determined by the disruption or introduction of an N-X-S/T glycosylation motif.

### Selection of escape mutants with deletions in the 220 loop

**Figure 3:**
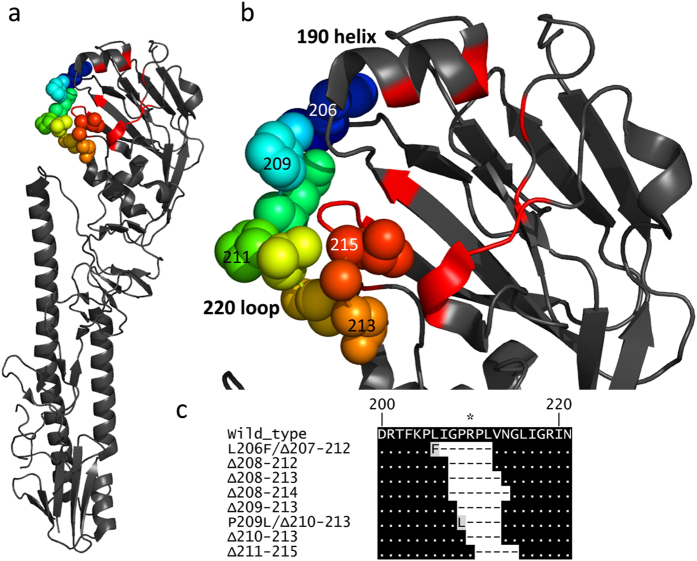
Location of 220 loop on the structure of H9HA on HA monomer (A) and HA1 head domain (B) (PDB, 1JSD). (**C**) Alignment of wild type UDL1/08 with deletion mutants. H9 numbering used throughout.

In addition to the escape mutants possessing single amino acid substitutions, mAbs EC12, CG12 and HA9, which had been shown to bind to antigen site H9-A, also selected for a total of eight unique escape mutants (L206F/Δ207–212, Δ208–212, Δ208–213, Δ208–214, Δ209–213, P209L/Δ210–213, Δ210–213, and Δ211–215) with deletions of between 4–7 amino acids within the 220 loop of HA, a region of the H9-A antigenic site in the vicinity of the receptor binding site. All deletions fell between the amino acids 207 and 215 with two individual mutants having additional substitutions, as well as the deletions, of L206F and P209L (Fig. 3). All deletion mutants showed a complete loss of detectable HI activity with the respective selecting mAbs (Table 3) but showed no observable reduction in their infectivity in MDCK cells and eggs (data not shown).

### Fine epitope mapping of antigenic sites H9-A and H9-B

To finely map the antigenic characteristics of proposed antigenic sites H9-A and H9-B, all escape mutants identified were reconstituted as described above. Antigenic cross reactivity of all 21 escape mutants with all seven of the mAbs with HI activity were then tested by HI assay (Table 4). All mutations in antigenic site H9-A and H9-B only affected the binding of mAbs that bound their respective antigenic sites; this further indicates that proposed antigenic sites H9-A and H9-B are discrete entities. The escape mutants located in antigenic site H9-A fell into two main clusters. Viruses with mutations at positions 212, 217, 234 and Δ220 were all able to escape from EC12, CG12, and HA9 but not JF7. Conversely the escape mutant T145I had reduced reactivity to JF7 but retained HI activity from mAbs EC12, CG12 and HA9. Escape mutants with substitutions at residue 183 showed different cross-reactivity profiles against the H9-A binding mAbs dependant on the specific amino acid present, whilst all three substitutions at 183 abolished binding by mAb JF7, N183S retained recognition by all other H9-A mAbs (EC12, CG12 and HA9), N183T moderately modulated the recognition of mAb EC12 as well as JF7 but not CG12 and HA9. Lastly, the N183D substitution reduced binding by all H9-A binding mAbs (EC12, CG12, HA9, and JF7), perhaps due to the large charge from the polar, uncharged asparagine, serine, or threonine to the negatively charged aspartic acid.

In site H9-B, the substitution R162W only affected binding of the selecting mAb, IB3 whilst the substitutions Q115R/P reduced the binding of both IB3 and JF8. Meanwhile changes at position 139 only affected the binding of JF8 whilst the escape mutations at position 120 prompted a reduction in the binding of both JF8 and ID2. This suggests that although ID2 and IB3 bind two discrete sites and still recognize each other’s escape mutants (although they must overlap enough to block each other’s binding as the epitope binning shows) JF8 appears to have a binding footprint that overlaps with each of the other H9-B binding mAbs.

Overall this data further supports the notion that proposed antigenic sites H9-A and H9-B are two entirely discrete antigenic sites which consist of smaller overlapping regions.

### Comparative bioinformatic analysis of identified escape mutant substitutions across H9N2 viruses

Next, we asked whether natural variations in sequenced field H9 influenza virus isolates occurred at the antigenic residues by this study by searching the GISAID database (Table 5). In addition we also looked specifically at H9N2 G1-like isolates for evidence of variation at these positions. Some of the identified residues had a wide variability, for example 162, 183, 212, 217 whilst other residues were fairly conserved for example at positions 115, 139, 145 and 234. This suggests that viruses with the variations at residues we identified as escape mutations may exist as naturally circulating antigenic variants in the field.

### Cross-reactivity of mAbs raised against UDL1/08 H9HA with divergent H9N2 viruses

To investigate the cross-reactivity of the UDL1/08 mAbs against other H9N2 influenza viruses we performed HI assays against a panel of closely and distantly related viruses using a constant concentration of 1 mg/ml of mAb (Table 6). No cross-reactivity was observed at all for the most distantly related H9N2 virus, A/turkey/Wisconsin/1/1966, or representative H5N1 and H7N1 viruses. The results indicate a general trend whereby the mAbs displayed higher cross-reactivity with more closely related viruses, as determined phylogenetically (see supplementary Fig. S3 online); however there were some exceptions that can be explained by natural variation at antigenic residues deduced in this work to be within the mAb epitopes. A/ck/Pak/UDL-02/08 has an alanine at position 120 in the H9-B antigenic site that likely accounts for its low reactivity with mAbs JF8 and ID2 (Table 7). A/Env/Bangladesh/10306/11 has a glutamine in place of an arginine at position 162 of H9-B and again this may explain its reduction in the binding to all H9-B group mAbs.

## Discussion

Substitutions in the hemagglutinin gene of influenza greatly influence virus antigenic properties giving rise to viruses with the ability to escape natural or vaccine-induced immunity and leading to vaccine failure in the field. This failure can result in viruses circulating and spreading unhindered in vaccinated animals^16^,^17^,^18^. To increase the effectiveness of vaccines more needs to be understood about the molecular factors that allow these viruses to emerge, as well as a better awareness of how a more potent, cross-protective immune response may be induced.

In this study we developed a novel and efficient way of generating mAbs against HA, with no prior mouse adaption of the virus required, through the use of viral vectors. An issue with generating avian influenza virus specific mAbs is that for the best antibody response, replicating virus is required. However, often H9N2 viruses are severely attenuated in mice, including the virus used for this study, UDL1/08 (James *et al*., unpublished, 2015), having little replicative activity and causing no weight loss^36^. Therefore immunization requires either mouse adaption of the virus, known to change the antigenic properties, or repeated inoculation, methods both used to varying success in previous studies^21^,^22^,^23^,^37^,^38^. By utilizing viral vectors known to infect and replicate efficiently in mice we were able to induce a potent immune response against unadapted H9HA, a method currently being researched for use in many human vaccines and viral and non-viral pathogens^39^.

We managed to generate a total of nine mAbs, seven of which showed neutralizing activity to infectious homologous virus (UDL1/08). Interestingly mAbs IG10 and HD8 showed no neutralizing activity by either HI or MNT at the highest concentrations tested (in excess of 2 mg/ml), a phenomenon observed in some previous studies^40^. Furthermore, IG10 showed no ability to block polyclonal antibody binding. Since both of these mAbs bound linear epitopes exposed by Western Blot (Fig. 1), we therefore speculate these mAbs bind to unfolded or misfolded protein. A further two mAbs, JF8 and ID2, displayed atypical neutralizing activity by MNT. This may be explained by the fact these mAbs bind peripherally to the receptor binding site and therefore are only partially neutralizing.

With our panel of mAbs we managed to select for a total of 26 different escape mutants with substitutions across 9 different residues, of these only position 183 and 212 have been identified previously as escape residues for H9N2 viruses, whereas positions 115, 120, 139, 145, 162, 217 and 234 are entirely novel^20^,^21^,^22^. Combining our epitope binning, competitive ELISA and escape mutant analysis, we determined that the seven mAbs occupied one of two antigenic sites that we designated H9-A and H9-B to discriminate them from previous H3 and H9 classification systems. Site H9-A shares some similarities to the previously described H9N2 antigenic site II, characterized on an H9HA from the Bei-like lineage, this includes the residue 183, and the residue 216, adjacent to our novel antigenic residues 212 and 217^22^. Antigenic site H9-B currently has no other H9 parallel though shows some similarity to the H3 antigenic sites A and D, although these amino acids are in a different conformation in the H9 hemagglutinin to that of H3 due to the truncation of the lateral/130 loop in the H9 structure^22^. Unlike previous studies we failed to find any evidence of the introduction or removal of potential glycosylation sites as a mechanism for escape in H9N2 viruses^19^,^22^,^23^. Utilising a competitive ELISA we showed that in pooled chicken polyclonal antisera from infected birds, H9-A was a moderately immuno-dominant epitope compared to H9-B. Interestingly, even when both antigenic sites were saturated a small percentage of around 20% of polyclonal antisera was still able to bind, we speculate some of this may be mAbs directed at NA, shown previously to illicit antibody responses in poultry^41^, or the stalk region of HA, as well as further epitopes in the head domain of HA not overlapping the H9-A and H9-B antigenic sites.

When tested against a selection of closely and distantly related H9N2 field isolates we found that there was a general trend towards higher cross reactivity with closely related viruses, as would be expected. There were a few exceptions to this correlation which we attributed to amino acid substitutions at antigenic residues bound by the effected mAbs, however this could not explain all the results, to give an example, even though A/chicken/Emirates/R66/2002 (R66) has the same amino acid residues at positions 115 and 162, the escape residues for mAb IB3, to UDL1/08, IB3 could not inhibit hemagglutination of R66. This indicates there must be further, undiscovered antigenic residues that we have not found when we generated escape mutants that contribute to this observed antigenic diversity. Studies utilising chimeric hemagglutinin molecules (with amino acids from one virus cloned into another) may be a strategy for teasing out further antigenic residues.

One of the escape mutants had a total of eight A to G transitions in the negative sense (see supplementary Fig. S2 online), leading to a total of four amino acid changes. By hemagglutinin inhibition assay with viruses engineered to harbour different combinations of the 4 different mutations we determined that only two of these amino acid changes were important for escape from the selecting mAb and speculated the remaining two were ‘passenger mutations’ (see supplementary Fig S2C online). Interestingly such targeted A to G transitions have been observed before in influenza genomes and been attributed to RNA editing mechanisms such as the activity of the ISG ‘adenosine deaminase acting on RNA-1′ or ADAR1^42^,^43^. ADAR1 deaminates adenosine nucleotides into inosine which, during replication, pairs with cytosine leading to an observed A to G transition. We speculate that RNA editing could have been involved in the generation of this particular escape mutant.

The mAbs CG12, EC12 and HA9, as well as selecting for escape mutants containing single amino acid substitutions, selected a group of variants with deletions in the 220 loop of the receptor binding site of hemagglutinin. There has previously been just a single report of escape mutants with receptor binding site deletions in a study looking at X-31, a lab adapted H3N2 virus^34^. The two selected H3N2 escape mutants both had an 8 amino acid deletion located deeper into the C terminus of the 220 loop than the viruses observed in our study. The H3N2 escape mutants also showed an altered binding profile to re-sialylated turkey red blood cells indicating these viruses had a change in receptor binding properties. Furthermore, a naturally occurring lineage of North American H7N2 viruses, isolated repeatedly from chickens, has also been identified as having a natural deletion in the 220 loop^44^. These viruses have been well characterized as having a slightly higher propensity for human-like receptor binding by glycan microarray and a higher rate of transmission to contact ferrets when compared to other circulating H7 viruses^33^,^45^. A single human case of the H7N2 virus has been reported^46^. Recently a genome announcement for a Chinese H9N2 was published which had a single amino acid deletion at position 216, this virus was described as showing a highly pathogenic phenotype when tested by IVPI, compared to a near isogenic isolate without the 216 deletion which was still low pathogenicity^4^. Overall these reports suggest that it is important to investigate the fitness and zoonotic potential of H9N2 and other avian influenza viruses with deletions in the 220 loop because, as well as being likely antigenic escape mutants, they may also have acquired changes in their receptor binding specificity and pathogenicity that may enhance their potentials as zoonotic agents.

To conclude, this study presents the discovery of two antigenic sites on G1-like H9N2 viruses. These antigenic sites contain seven novel variant amino acid residues that should be treated as antigenic markers for vaccine matching with circulating field strains when considering the most suitable vaccine seed strain, either veterinary or human pandemic potential. As well as this, understanding the basis of antigenicity of these viruses will help virus surveillance efforts determine that antigenic variants are emerging or prevalent in a population.

## Methods

### Ethics Statement

All animal studies and procedures were carried out in strict accordance with the guidance and regulations of European and United Kingdom Home Office regulations under project licence numbers 30/2683 and 30/2952. As part of this process the work has undergone scrutiny and approval by the ethics committee at The Pirbright Institute.

### Viruses and cells

Using standard reverse genetics an H9N2 virus of the G1 lineage, A/chicken/Pakistan/UDL-01/2008 (UDL1/08), was rescued from bidirectional reverse genetics plasmids as previously described^47^. All other H9N2, H5N1 or H7N1 viruses (including stocks of UDL1/08 and its escape mutants used for downstream antigenic analysis) used were rescued using the HA and NA of the named viral strain with the remaining gene segments from A/Puerto Rico/8/1934 (PR8). Multiple, independently rescued virus stocks of UDL1/08 were propagated in 10 day old specific pathogen free (SPF) embryonated chicken eggs and titrated by plaque assay or TCID_50_ on MDCKs. To guarantee the genetic purity of all the escape mutants used for downstream antigenic analysis identified amino acid substitution in HA were reintroduced into the reverse genetics plasmids by site directed mutagenesis and mutants were reconstituted through virus rescue as H9N2:PR8 2:6 recombinants. Full length sequencing (see below) of the HA and NA gene segments of all reconstituted escape mutants was performed to verify no additional mutations or reversions appeared after rescue of the reconstituted escape mutants. Viruses were purified by ultracentrifugation through a continuous 30–60% w/v sucrose gradient.

MDCK cells were maintained in Dulbecco modified Eagle medium (DMEM) supplemented with 10% fetal bovine serum (FBS) at 37^o^C, 5% CO_2_. Human embryonic kidney 293T (293T) cells were maintained in DMEM containing 2% FBS and 1 mM sodium pyruvate at 37^o^C, 5% CO_2_.

### Production and purification of recombinant HA glycoprotein

Recombinant HA glycoprotein was produced and purified using Drosophila Expression System (DES®, Life technologies) as described previously^48^. Briefly, the HA gene of UDL1/08, lacking the transmembrane domain and signal peptide, was cloned into DES expression vector (pMT-BiP-V5-His). The recombinant HA expression plasmid was co-transfected into S2 (drosophila) cells together with a hygromycin B resistance plasmid (pCoHYGRO, Life technologies). Individual S2 cell clones expressing recombinant HA were selected after hygromycin B treatment. Recombinant HA was secreted into culture supernatant after CuSO_4_ induction and then purified by affinity chromatography using a Chelating Sepharose Fast Flow column (GE Healthcare Life Sciences). The purity of recombinant HA was then confirmed using SDS-PAGE and Western Blot.

### Generation of vaccine for mAb production

Modified vaccinia Ankara (MVA) and human adenovirus 5 (Ad5) vectors were used as vaccines for immunization of Biozzi mice. Both vaccines were generated at the Viral Vector Core Facility at the Jenner Institute (Oxford, UK). Briefly, the recombinant MVA vaccine expressing H9HA of UDL1/08 (rMVA-H9HA) was generated through cloning the coding region of UDL1/08 HA into the MVA shuttle vector (MVA-GFP-TD). The resulting shuttle vector and a bacterial artificial chromosome (BAC) containing the integrated MVA genome were co-transfected into primary chicken embryo fibroblasts (CEFs). Through recombination the rMVA-H9HA was completed when the HA gene was integrated in the thymidine kinase locus of the MVA genome downstream of the vaccinia virus P7.5 promoter^49^.

Recombinant Ad5 expressing UDL1/08 H9HA (rAd5-H9HA) was generated by cloning the HA gene of UDL1/08 into the Ad5 shuttle vector (pENTR4-LPTOS) and co-transfecting into 293 HEK cells with the plasmid containing the Ad5 genome lacking the E1 and E3 regions. The HA gene insertion was directed into the E1 deleted region and expression of H9HA was under the control of human cytomegalovirus immediate early promoter^50^.

Titration of rMVA-H9HA and Ad5-H9HA was undertaken by performing plaque/foci forming assays on CEFs and 293 HEK cells respectively.

### Mouse immunization

Antibody responses against HA of UDL1/08 were induced in two adult Biozzi ABH mice (Harlan^®^). Vaccination consisted of an initial subcutaneous (SC) inoculation of 5000 pfu in 100 μL of recombinant adenovirus H9HA (rAd-H9HA). Twenty one days later, each mouse was boosted with 5000pfu (in 100 μL) of MVA H9HA (rMVA-H9HA) via the SC route. Forty eight days after the rMVA-H9HA boost, each mouse was additionally boosted with 5 μg (in 100 μL) of recombinant H9HA purified protein (produced in S2 cells) via the intravenous route. Four days later mice were humanely euthanized and splenocytes were harvested for fusion with myeloma cells. Hybridoma cells expressing mAbs against H9HA were selected by ELISA against purified UDL1/08 virus adopting a standard mAb screening method^51^.

Hybridoma stocks were grown in suspension using IgG-depleted serum using the miniPERM Bioreactor (Sarstedt). Monoclonal antibodies in the miniPERM supernatant were then purified by affinity purification using a HiTrap Protein G HP column (GE healthcare) and dialysed into PBS.

### Characterization of monoclonal antibodies

Isotyping of mAbs was carried out using the Isostrip Mouse Monoclonal Antibody Isotyping Kit (Roche). Hemagglutination inhibition titres were determined using a stock solution of 1 mg/ml of mAb, determined by spectroscopy and confirmed by SDS gel electrophoresis, as previously described (4HA units/25 uL, 1% chicken red blood cells in PBSa)^52^. Microneutralization assays were performed in quadruplicate using a standard solution of 1 mg/ml of monoclonal antibody as described elsewhere^52^. Linearity of epitopes was determined by running an excess of purified UDL1/08 virus against an excess of mAb on a standard reducing Western blot, presence of a band indicated the epitope recognised was linear. Positive control polyclonal anti-H9HA chicken antiserum was generated by immunizing chickens with rAd-H9HA in a similar manor to mice as detailed above.

### Selection of escape mutants

Escape mutants of UDL1/08 were selected in a single step protocol similar to that previously described^20^. Firstly, between 10^5^–10^7^pfu of virus stock rescued from reverse genetics plasmids and grown up in eggs for 1 passage was incubated at 37 ^o^C for 2 hours with a saturating concentration of purified mAb. Virus was then serially diluted in MDCKs or 10 day old embryonated chicken eggs. Virus was harvested and the highest infectious dilution (judged by CPE if from MDCKs or hemagglutinination assay if from egg allantoic fluid) was again incubated with a saturating concentration of monoclonal antibody and passaged again in cells or eggs.

### Sequencing and PCR

The sequence analysis of HA gene was performed as described previously^53^, briefly viral RNA was extracted from allantoic fluid or cell culture supernatant using the QIAamp Viral RNA Mini kit (Qiagen). Reverse transcription was performed using the Verso cDNA synthesis kit (Thermo Scientific) and the universal FluA primer (sequence: AGCAAAAGCAGG). PCR was performed using UDL1/08 HA1 specific primers containing M13F and M13R sequences at the 5′ and 3′ ends respectively (sequences available on request). Resulting products were purified using the QIAquick PCR purification kit (Qiagen) and Sanger sequenced with M13F and M13R primers. Sequences were analyzed, assembled and aligned using VectorNTI (Life Technologies™).

### Competitive ELISA

Competitive ELISA was carried out by first coating each well of a maxisorp ELISA plates (Thermo Fisher Scientific) with 200 ng purified UDL1/08 virus diluted in 100 μL of carbonate buffer (pH 9.6) overnight at 4^o^C. Virus coated plates were blocked at room temperature with 5% milk powder (Marvel) in PBS-tween 0.05% (PBS-T) for 2 hours at room temperature. Plates were washed thrice with wash buffer (PBS-tween 0.05%). To block polyclonal antisera binding to the mAb binding sites of hemagglutinin a saturating concentration (4 ug/ml per antibody) was made up in 100 μL of PBS-T, 2% Marvel and incubated at room temperature for 1 hour. Plates were again washed thrice. To prove remaining antibody binding sites of haemagglutinin were not blocked by the mAbs, excess pooled post-infection chicken sera (1/250 dilution) raised against UDL1/08 was made up in 100 μL of PBS-T, 2% Marvel and added to all wells for 1 hour at room temperature. Plates were once again washed thrice and HRP conjugated goat anti-chicken IgY H + L (Southern Biotech 6100-05) was added in 2% Marvel in PBS-T at a dilution of 1/4000 for 45 minutes. Plates were washed x4 with PBS-T and once with PBS then 100 uL of TMB substrate (BD biosciences) was added for 10 minutes. The reaction was stopped using 2 M H_2_SO_4_. A_450_ was then read on an ELISA plate reader. The average value of wells with no monoclonal antibodies added was taken as 0% inhibition; this was confirmed with a control, non-neutralising mAb (IG10). The average background absorbance of wells with no polyclonal antisera added was taken as the negative control of 100% inhibition.

### Biophysical techniques

Epitope binning and determination of dissociation constants was performed on the Octet Red interferometer (Pall Fortebio) using anti-mouse Fc capture (AMC, 18-5090) as previously described^54^,^55^. All experiments were carried out in HBS-EP buffer (10 mm HEPES (pH 7.4), 150 mm NaCl, 3 mM EDTA, 0.005% (v/v) Tween 20).

For epitope binning the first antibody was loaded in excess (>1.5 uM) onto the AMC biosensors, the sensors were next exposed to purified recombinant UDL1/08 HA protein at a concentration of 100 nM, finally the sensors were exposed to competing mAbs at a concentration of 600 nM. The negative control consisted of the same antibody used as both first and second mAbs, the reading from this biosensor was subtracted from all other reads. Any antibody binding at a substantially higher rate than the negative control would be counted as positive binding and therefore interpreted as binding a different epitope. All 7 neutralizing antibodies were tested in every possible combination against each other.

Dissociation constants were determined by binding each mAb to a row of AMC biosensors at a concentration of 25 nM. Next a 2 fold dilution of HA starting at 1 μM was bound to the antibodies. Offset values were plotted against concentration and modelled to one site specific binding equation using Graphpad Prism 6 to estimate the K_D_ values.

### Bioinformatic analysis of amino acid distributions/site and Structures

For bioinformatic analysis all H9 HA amino acid sequences (as of August 2015) were downloaded from the NCBI Influenza Virus Database (http://www.ncbi.nlm.nih.gov/genomes/FLU/aboutdatabase.html) and aligned using MEGA version 6.0^56^. The distribution of amino acids at each antigenic residue was then assessed.

All structural images were generated and rendered in Pymol (Schrödinger) using the structure PDB-ID:1JSD (H9HA from A/swine/Hong Kong/9/98)^57^.

## Additional Information

**How to cite this article**: Peacock, T. *et al.* Antigenic mapping of an H9N2 avian influenza virus reveals two discrete antigenic sites and a novel mechanism of immune escape. *Sci. Rep.* **6**, 18745; doi: 10.1038/srep18745 (2016).

## Supplementary Material

Supplementary Figures 1, 2 and 3

## Figures and Tables

**Table 1:** Properties of anti-H9N2 mouse mAbs.

mAb	HI titre/mg	MN titre/mg	Isotype IgH/Light chain	Linear/non- linear	K_D_ (nM)	Proposed antigenic site^a^
CG12	5120	3620	IgG2a/κ	Non linear	95.6	H9-A
EC12	2560	3050	IgG2a/κ	Non linear	63.8	H9-A
HA9	5120	5120	IgG2a/κ	Non linear	76.1	H9-A
JF7	5120	5120	IgG2a/κ	Linear	21.1	H9-A
JF8	2560	ND^b^	IgG2a/κ	Non linear	5.3	H9-B
ID2	2560	ND^b^	IgG2b/κ	Linear	34.2	H9-B
IB3	5120	3620	IgG2a/κ	Linear	41.5	H9-B
IG10	n/a^c^	n/a	IgG2a/κ	Linear	n/a	n/a
HD8	n/a	n/a	IgG2a/κ	Linear	n/a	n/a

^a^Antigenic site was determined by epitope binning against purified protein on the octet, blocking mAbs were grouped together into two bins called H9-A and H9-B.

^b^Not determined due to inconsistent and non-concentration dependant neutralization ability.

^c^Not applicable due to lack of neutralizing activity.

**Table 2:** mAb selected escape mutants with amino acid substitutions in the HA1 region of hemagglutinin.

mAb ID	Amino acid substitution in escape mutant^a^	Antigenic Site	Reduction in HI titre (log2)
EC12	N183T,	H9-A	1
	N183D		>7
	L212P & I217T^b^,		3 (1 + 2 individually)^b^
CG12	R234Q		2
JF7	N183S,		>6
	T145I		3
ID2	R139M,	H9-B	<1
	T120K		2
JF8	R139M,		5
	R139G		6
IB3	Q115R,		5
	Q115P,		>6
	R162W		3

^a^H9 numbering used throughout.

^b^Selected escape mutant had additional mutations that were determined structurally and by HI to be antigenically irrelevant for the escape mutant (see supplementary Fig. S2 online).

**Table 3:** mAb selected escape mutants with amino acid deletions in the 220 loop region of HA1.

mAb ID	Escape mutant^a^	Reduction in HI titre (log2)
EC12	Δ208–214	>7
	Δ209–213	>7
	P209L/Δ210–212	>7
	Δ210–213	>7
	Δ211–215	>7
CG12	Δ208–212	>7
	Δ208–213	>7
	Δ209–213	>7
	Δ211–215	>7
HA9	L206F/Δ207–212	>6
	Δ208–212	>6
	Δ211–215	>6

^a^H9 numbering was used throughout.

**Table 4:** Cross-reactivity of H9N2 escape mutants with mAbs.

Selecting mAb	Antigenic Site	Mutation	Reduction in HI titre by escape mutant						
			H9-A				H9-B		
			EC12	CG12	HA9	JF7	ID2	JF8	IB3
		Wild type	—a	—	—	—	—	—	—
EC12/HA9/CG12	H9-A	Δ220^b^	**>7**^c^	**>7**	**>7**	—	—	—	—
EC12		L212P & I217T	**3**	2	2	—	—	—	—
		N183D	**>7**	>7	>7	>7	—	—	—
		N183T	**1**	—	—	>7	—	—	—
CG12	H9-A	R234Q	1	**2**	3	—	—	—	—
JF7	H9-A	N183S	—	—	—	**>7**	—	—	—
		T145I	—	—	—	**3**	—	—	—
ID2/JF8	H9-B	R139M/G^d^	—	—	—	—	—	**>5**	—
		T120K	—	—	—	—	**1**	>5	—
IB3	H9-B	Q115R/P	—	—	—	—	—	4	**6**
		R162W	—	—	—	—	—	—	**3**

^a^Indicates no change in HI titre compared to the wild type virus. Numbers indicate log_2_ change in HI titre relative to wild type when mutation is present.

^b^Δ220 indicates all 8 escape mutants with deletions in the 220 loop, all 220 loop deletion viruses gave same binding profile so are shown as a single row.

^c^numbers in bold indicate escape mutants and their selecting mAbs.

^d^R139M/G indicated both R139M and R139G gave the same reactivity profile to panel of mAbs.

**Table 5:** Bioinformatic analysis of natural H9HA mutations.

Amino acid residue^a^	Equivalent antigenic sites in H3/H9 subtypes	Amino acid substitution	Selecting mAb(s)	Total occurrence in H9N2 taken from GISAID database (%)^b^	Occurrence in G1-like only from GISAID database (%)^c^
115	A/-	Q- > R/P	IB3	Q (98.1), R (<0.1), P (0)	Q (99.5), R (0), P (0)
120	−/−	T- > K	ID2	T (87.7), K (0.7)	T (86.9), K (0)
139	−/−	R- > G/M	JF8, ID2	R (99.0), G (0.3), M (<0.1)	R (100), G (0), M (0)
145	B/-	T- > I	JF7	T (99.4), I (<0.1)	T (99.5), I (0.3)
162	D/-	R- > W	IB3	R (42.5), W (1.6)	R (90.6), W (0)
183	B/Site II	N- > S/T/D	JF7, EC12	N (80.6), S (1.5), T (0.8), D (16.4)	N (95.1), S (0.8), T (2.8), D (0.8)
212	−/−	L- > P	EC12	L (98.3), P (0.2)	L (99.5), P (0.3)
217	−/−	I- > T	EC12	I (13.8), T (0.8)	I (73.1), T (4.4)
234	D/-	R- > Q	CG12	R (99.4), Q (0.2)	R (99.7), Q (0)

^a^H9 numbering.

^b^A total of 3499 H9 HA1 sequences were downloaded from the NCBI Influenza Database (as of 26 August 2015) for analysis.

^c^A total of 383 G1-like sequences were downloaded from the NCBI Influenza Database (as of 26 August 2015) for analysis.

**Table 6:**
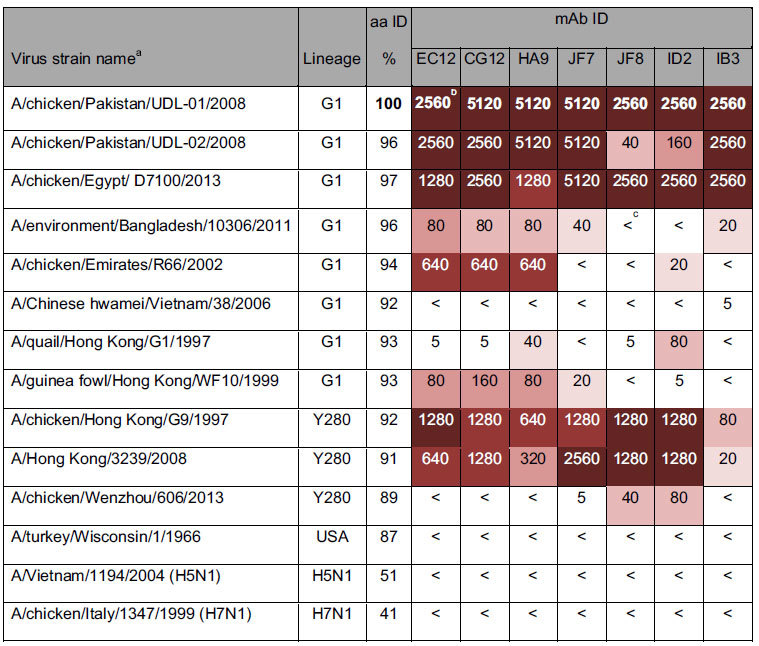
Cross-reactivity of mAbs against closely related and divergent H9N2 viruses.

^a^Unless otherwise specified, all viruses listed are of the subtype H9N2. ^b^Numbers in bold indicate homologous titers. ^c^<indicated HI titers of <5. Colors show degree of difference between homologous titer and other titers with each change in shade indicating a 4-fold reduction.

**Table 7:** Amino acid identities at antigenic positions amongst selected H9N2 isolates.

Virus	Amino acid residue at position^a^								
	115	120	139	145	162	183	212	217	234
A/chicken/Pakistan/UDL-01/2008	Q	T	R	T	R	N	L	I	R
A/chicken/Pakistan/UDL-02/2008	—b	A	—	—	—	—	—	—	—
A/chicken/Egypt/D7100/2013	—	—	—	—	—	—	—	—	—
A/environment/Bangladesh/10306/2011	—	—	—	—	Q	—	—	—	—
A/chicken/Emirates/R66/2002	—	—	—	—	—	—	—	L	—
A/Chinese hwamei/Vietnam/38/2006	—	—	—	—	—	—	—	Q	Q
A/quail/Hong Kong/G1/1997	—	—	—	—	—	—	—	Q	—
A/guinea fowl/Hong Kong/WF10/1999	—	—	—	—	—	—	—	Q	—
A/chicken/Hong Kong/G9/1997	—	—	—	—	—	—	—	Q	—
A/Hong Kong/3239/2008	—	—	—	—	—	—	—	Q	—
A/chicken/Wenzhou/606/2013	—	—	—	—	Q	D	—	M	—
A/turkey/Wisconsin/1/1966	—	K	—	—	E	D	—	—	—

^a^H9 numbering.

^b^indicates no change from A/chicken/Pakistan/UDL-01/2008.
