# Supplementary material for: Antigenic mapping of an H9N2 avian influenza virus reveals two discrete antigenic sites and a novel mechanism of immune escape
Source: Sci Rep. 2016 Jan 7;6:18745. doi: 10.1038/srep18745 (PMC4704030; doi:10.1038/srep18745)
Supplement: Supplementary Figures 1, 2 and 3 [file srep18745-s1.pdf]

Supplementary Figures

Antigenic mapping of an H9N2 avian influenza virus reveals two discrete antigenic sites and a novel mechanism of immune escape

Thomas Peacock[a,b], Kolli Reddy[a], Joe James[a,b], Beata Adamiak[a], Wendy Barclay[b], Holly Shelton[a] and Munir Iqbal[a]*

[a]The Pirbright Institute, Pirbright, Woking, UK [b]Imperial College London, UK

*Corresponding author:

Dr Munir Iqbal,

The Pirbright Institute,

Pirbright,

Woking,

GU24 0NF

United Kingdom

E-mail. munir.iqbal@pirbright.ac.uk

Tel: +44 (0)1635-577273

Running title: H9N2 hemagglutinin antigenic mapping

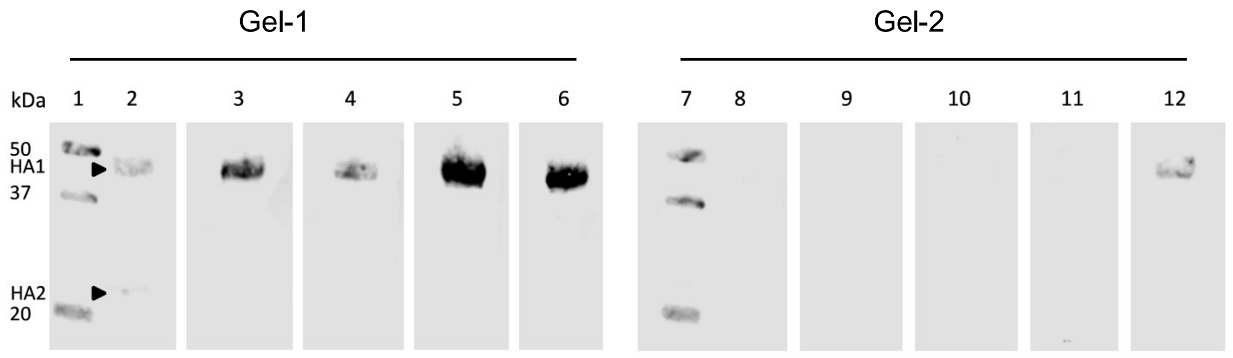

Fig S1. Western blot analysis of different monoclonal antibodies against purified H9N2 (UDL1/08) virus. Proteins of purified virus were separated on a 4-12% Bis-Tris PAGE gel-1 and gel-2. Molecular weight marker: lanes 1 and 7. Purified virus protein: lanes 2-6 and 8-12. The virus HA glycoprotein was probed with primary antibodies: lane 2, positive control polyclonal anti-H9HA antiserum raised by immunizing chickens with rAd-H9HA vaccine expressing HA glycoprotein of UDL1/08 virus; lane 3, mAb IB3; lane 4, mAb ID2; lane 5, mAb HD8; lane 6, mAb IG10; lane 8, mAb JF8; lane 9, mAb CG12; lane 10, mAb EC12, lane 11, mAb HA9 and lane 12, mAb JF7.

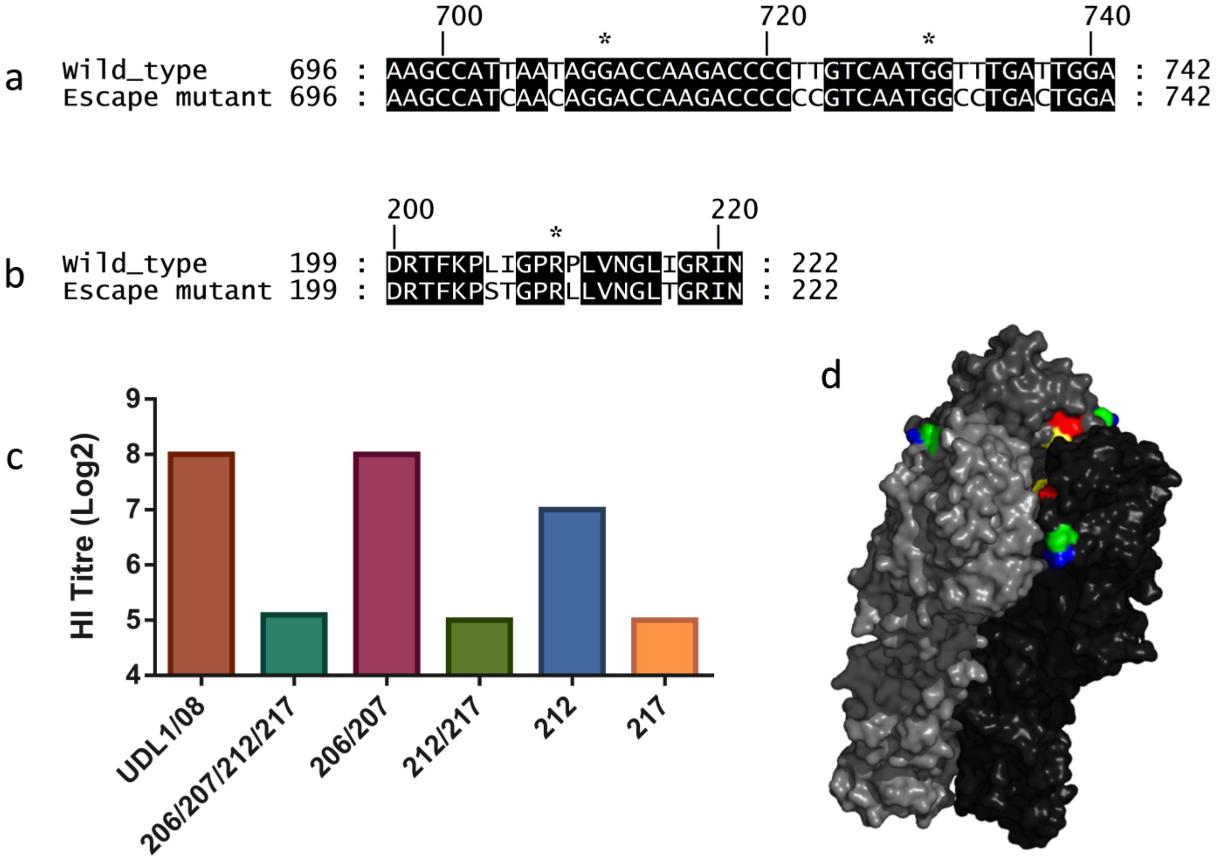

Fig S2. Determination of antigenic residues from an escape mutant with multiple amino acid changes. Alignments of nucleotide (A) and amino acid (B) sequences of multiple amino acid escape mutant. C) HI titres of mAb EC12, the selecting mAb, against individual amino acid changes in the escape mutant. D) Structural location of amino acid changes on H9HA trimer (PBD, 1JSD), positions 206 in yellow, 207 in red, 212 in blue and 217 in green (H9 numbering used throughout).

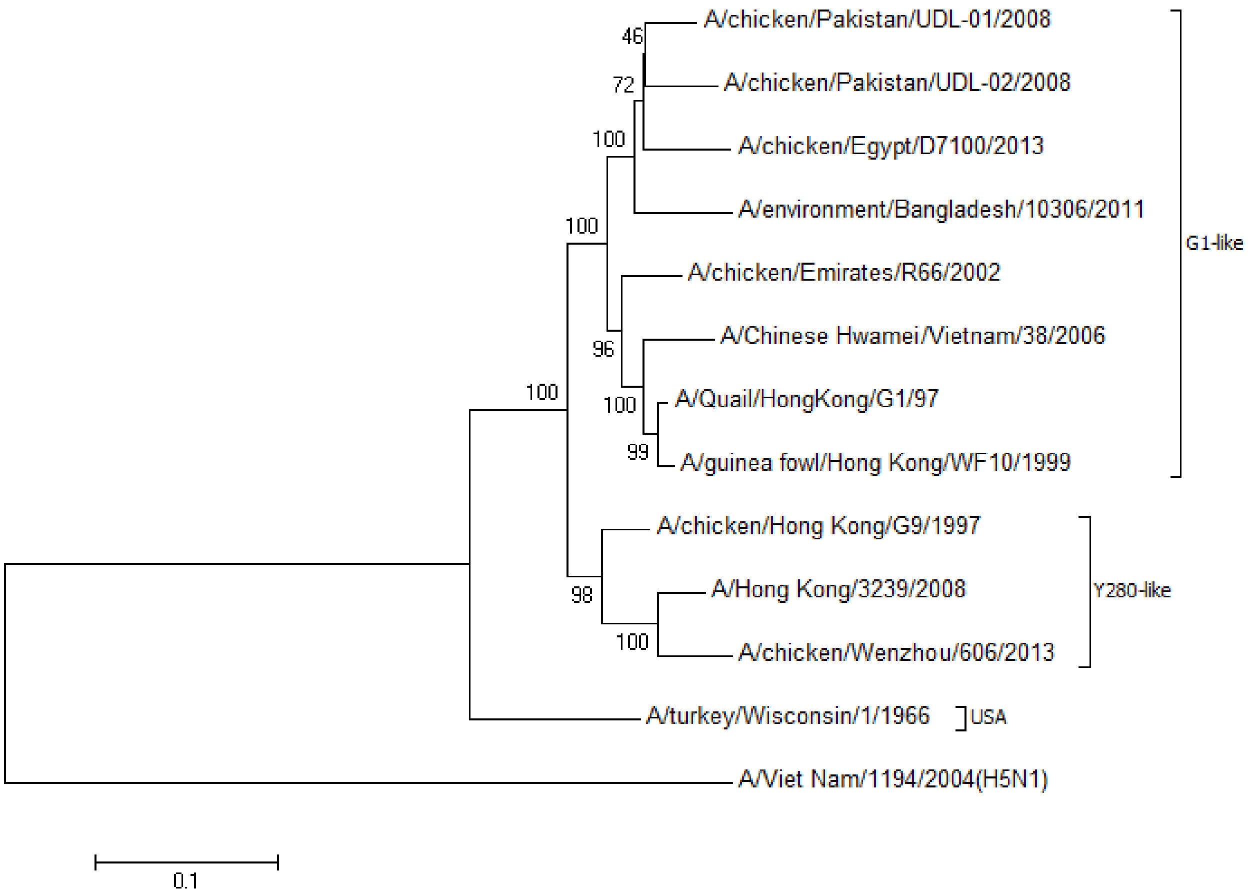

Fig S3. Phylogenetic tree of the H9 HA genes of viruses used in this study. Nucleotide coding regions of the HA genes were aligned with MEGA version 6.06 using a neighbour joining method. Values at each node indicated the confidence value from a bootstrap analysis with 1000 replications.
